# Left Atrial Isomerism in a Four-Year-Old Girl Diagnosed due to Gastrointestinal Bleeding: A Case Report

**DOI:** 10.7759/cureus.104846

**Published:** 2026-03-08

**Authors:** Ken Matsushita, Yoichi Iwamoto, Hirotaka Ishido, Naomi Ino, Yuta Uchida, Kohei Osada, Yoshio Sakurai, Satoshi Masutani

**Affiliations:** 1 Pediatrics, Saitama Medical Center, Saitama Medical University, Kawagoe, JPN; 2 Pediatric Intensive Care, Saitama Medical Center, Saitama Medical University, Kawagoe, JPN

**Keywords:** atrioventricular septal defect, duplicated inferior vena cava, esophageal and gastric varices, heterotaxy syndrome (hs), hypovolemic shock, polysplenia

## Abstract

Heterotaxy is characterized by the presence of multisystem comorbidities, some of which may result in fatal outcomes. However, diagnosing left atrial isomerism (LAI), a form of heterotaxy, at an early age can be challenging in the absence of typical findings.

A four-year-old girl presenting with hypovolemic shock resulting from esophageal variceal bleeding was transported to Saitama Medical Center, Japan, and was diagnosed with LAI post-admission. LAI had not been diagnosed prior to her hospital admission, even though she underwent intracardiac repair for atrioventricular septal defect at 10 months of age. The condition of the patient was characterized by the presence of a duplicated inferior vena cava (IVC), and not by an IVC defect, which is typically associated with LAI. The prevalence of duplicated IVCs in patients with heterotaxy remains unclear. Duplicated IVCs can be detected by abdominal ultrasonography, and an understanding of anatomical variations in the venous pathway may be helpful for the early diagnosis of heterotaxy.

## Introduction

Atrioventricular septal defect (AVSD) is one of the congenital heart diseases that originates from malformation of the endocardial cushion [1]. It is characterized by the absence of the atrioventricular septum with a common atrioventricular valve orifice [1]. These structural characteristics result in increased pulmonary blood flow and lead to heart failure in the clinical course of the disease [2].

AVSD has been documented in 75% of cases in conjunction with extracardiac anomalies. Among these cases, 15% of patients diagnosed with AVSD were found to have heterotaxy [3]. Heterotaxy is defined as a condition in which the internal thoracoabdominal organs demonstrate an abnormal arrangement across the left-right axis of the body [4]. It occurs in approximately one in 10,000 live births and is classified into two categories: left and right isomerism [5]. Left atrial isomerism (LAI) is characterized by bilateral left-sided morphology. Representative cardiac anomalies in LAI include AVSD and inferior vena cava (IVC) defects. In contrast, right atrial isomerism (RAI) is characterized by bilateral right-sided morphology. A functionally univentricular heart and absence of the spleen are characteristic anomalies associated with RAI [6,7]. Intestinal malrotation is a common extracardiac anomaly in heterotaxy syndrome and occurs in both RAI and LAI.

LAI is notable for the presence of additional extracardiac comorbidities, including polysplenia, intestinal malrotation, and an abnormal portal vein course [8,9]. Some complications in LAI, such as bacterial infection resulting from immunodeficiency, splenic dysfunction, or gastrointestinal bleeding, may result in fatal outcomes [10]. Therefore, it is imperative to consider LAI in the differential diagnosis of patients with AVSD.

We encountered a case of LAI diagnosed in a four-year-old girl who developed hypovolemic shock due to ruptured esophageal varices years after undergoing intracardiac repair for AVSD in infancy. We describe this case and discuss the factors contributing to the difficulty in the early diagnosis of LAI.

## Case presentation

The patient was a four-year-old girl. She was delivered at a medical facility other than Saitama Medical Center, Japan. On day three of life, she developed tachypnea and was diagnosed with AVSD. She underwent pulmonary artery banding and patent ductus arteriosus ligation on day four of life. At 10 months of age, she underwent a modified one-patch repair for AVSD. Moderate aortic valve regurgitation persisted postoperatively, and she subsequently underwent aortic valvuloplasty on postoperative day seven. Following this series of operations, her condition stabilized, and she was referred for outpatient follow-up.

Three days prior to her visit to our hospital at the age of four, she experienced abdominal pain. The pain was located in the umbilical region and exacerbated after meals. Two days prior to her visit to our hospital, she developed a cough, runny nose, and several episodes of melena. One day prior to her visit to our hospital, she exhibited a fever of 38.9℃. Consequently, she was unable to walk by herself and was transferred to our hospital.

Her body temperature was 38.1℃, heart rate was 123 beats per minute, blood pressure was 101/60 mmHg, respiratory rate was 24 breaths per minute, and peripheral oxygen saturation was 99% in room air. The results of her physical examination were notable for pallor of the skin, a pale palpebral conjunctiva, cold extremities, and umbilical tenderness. Capillary refill time was less than 1 s.

A laboratory blood examination revealed severe anemia, a hemoglobin (Hb) level of 3.9 g/dL, a platelet (Plt) count of 179000/μL, and preserved coagulation function. A rapid antigen test for influenza A was positive (Table 1).

**Table 1:** Initial laboratory findings on admission The blood test reveals severe anemia and a slight coagulation abnormality. WBC: white blood cell; RBC: red blood cell; Hb: hemoglobin; Plt: platelet; PT: prothrombin time; APTT: activated partial thromboplastin time; AST: aspartate aminotransferase; ALT: alanine aminotransferase; T-Bil: total bilirubin; BUN: blood urea; Alb: albumin; Cre: creatinine nitrogen

Parameter	Value	Reference range	Unit
WBC	7400	4200-18300	/μL
RBC	1.93	405-530	10^6^/μL
Hb	3.9	11.1-14.2	g/dL
Plt	179	18.0-58.0	10^3^/μL
PT	10.7	9.6-13.1	s
APTT	19.4	24-34	s
D-dimer	0.40	<1.00	μg/mL
Alb	2.8	3.5-4.7	g/dL
AST	26	24-44	U/L
ALT	14	9-30	U/L
T-Bil	0.4	0.3-0.9	mg/dL
BUN	14	5.5-19.3	mg/dL
Cre	0.22	0.2-0.39	mg/dL
Influenza A	Positive		

**Figure 1:**
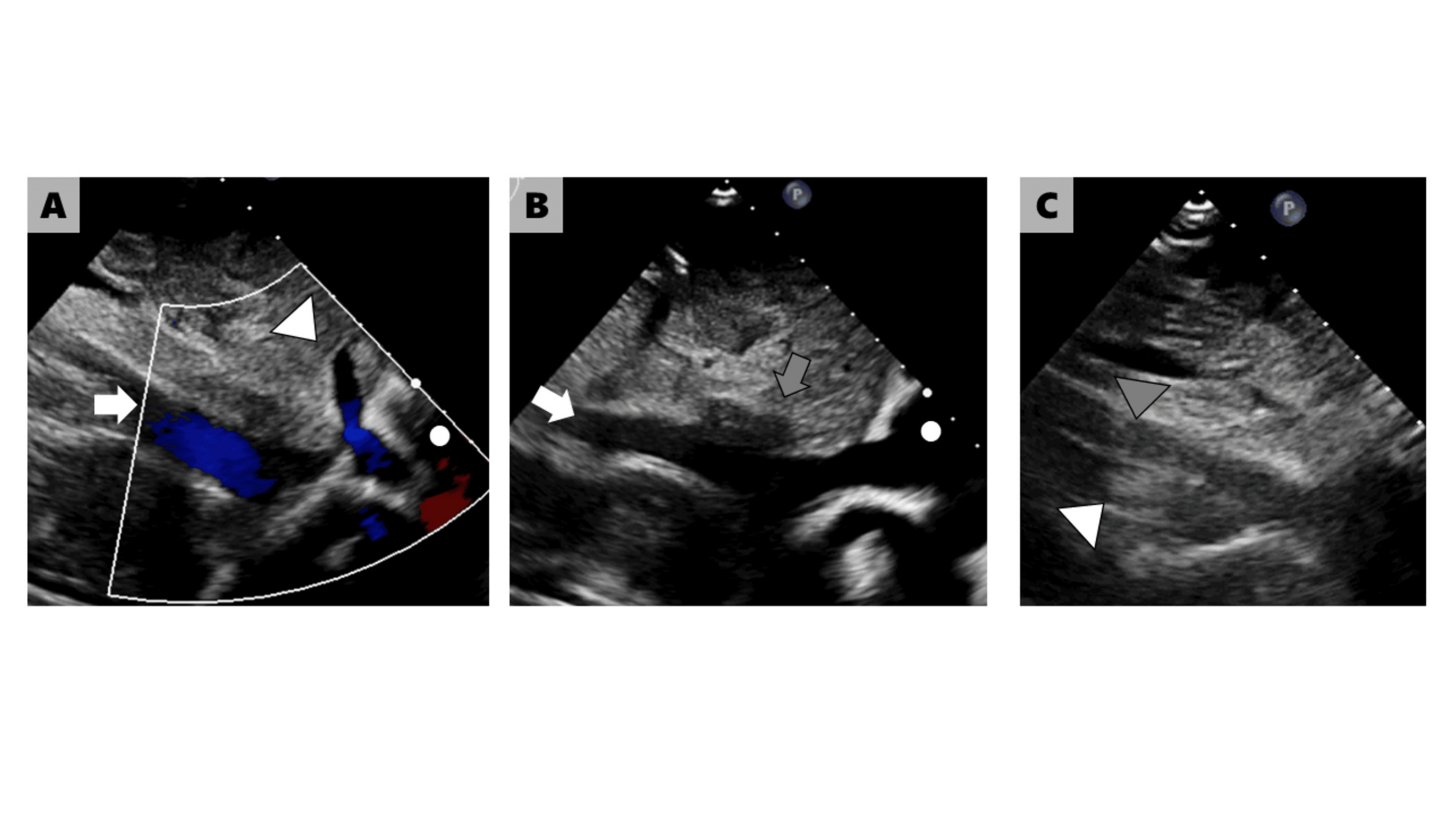
Transthoracic echocardiography A. The IVC (arrow) enters the right atrium (circle) without any IVC defect. The hepatic vein (arrowhead) connects to the IVC. B and C. The IVC (arrow) bifurcates (gray arrow) caudal to the hepatic vein inflow. On CT, these represent the right IVC (white arrowhead) and the left IVC (gray arrowhead). IVC: inferior vena cava; CT: computed tomography

Transthoracic echocardiography showed a balanced four-chamber heart, and her left ventricular ejection fraction was 74%. There was trivial tricuspid and aortic regurgitation. Her IVC drained into the right atrium (Figure 1).

**Figure 2:**
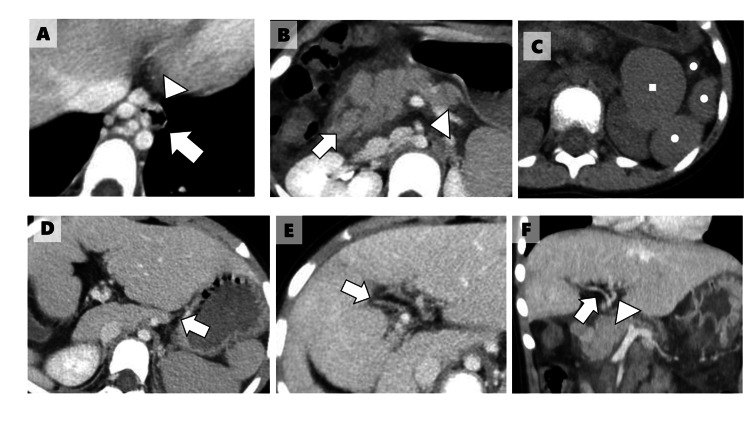
Dynamic contrast-enhanced CT A. Esophageal varices extend from the thoracic to the abdominal esophagus. No extravasation of contrast agent is observed (arrow: esophagus; arrowhead: esophageal varix). B. The horizontal part of the duodenum is located far from the superior mesenteric artery, indicating intestinal malrotation (arrow: horizontal part of the duodenum; arrowhead: superior mesenteric artery). C. Multiple spleens are observed (circle: spleen; square: stomach). D. Agenesis of the pancreatic tail is observed (arrow: pancreatic tail). E and F. The portal vein is occluded just proximal to its entry into the liver (arrow: portal vein; arrowhead: hepatic artery). The intrahepatic portal vein is poorly enhanced. CT: computed tomography

**Figure 3:**
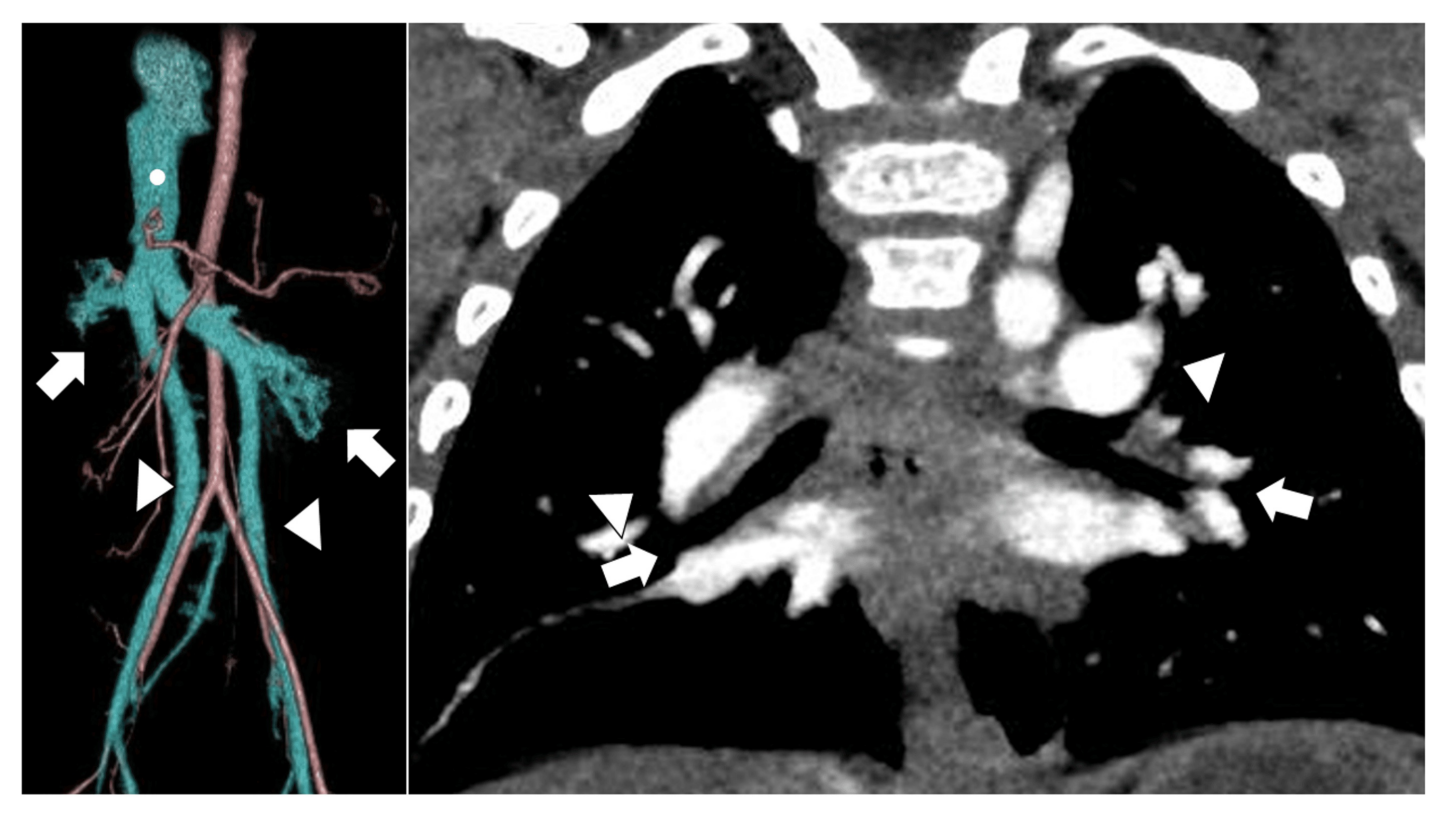
Vascular structures on CT A: The left and right renal veins (arrow) drain separately into the left and right iliac veins (arrowhead). The iliac veins subsequently converge to form a single IVC (circle). B: The CT image was obtained at the time of intracardiac repair. It shows that both main bronchi (arrow) pass below their corresponding pulmonary arteries (arrowhead). CT: computed tomography; IVC: inferior vena cava

A dynamic contrast-enhanced computed tomography (CT) scan was performed to identify the source of gastrointestinal bleeding. The scan revealed esophageal varices extending from the thoracic to the abdominal portion of the esophagus (Figure 2A). A focal obstruction of the portal vein was observed just proximal to its entry into the liver, accompanied by collateral vessels supplying the esophageal varices. Additionally, intestinal malrotation, multiple spleens, and agenesis of the pancreatic tail, typical findings of LAI, were noted (Figures 2B, 2C, 2D). The left and right renal veins drained separately into the left and right iliac veins, respectively, forming the left and right IVCs. These vessels subsequently converged to form a single IVC (Figure 3A). We obtained the CT images that had been taken at the previous hospital before she underwent intracardiac repair. These images revealed that both main bronchi passed below their corresponding pulmonary arteries (Figure 3B).

**Figure 4:**
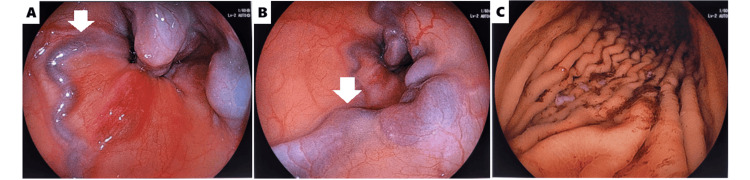
Upper gastrointestinal endoscopy A (esophagogastric junction) and B (mid-esophagus): Esophageal varices (arrow) extending from the mid-esophagus to the esophagogastric junction are observed. No obvious active bleeding is seen. C (greater curvature of the stomach): Black gastric residual material is present in the stomach.

The patient was diagnosed with LAI based on the presence of AVSD, multiple spleens, and bilateral hyparterial bronchial branching pattern, according to Berg’s definition [11]. The esophageal varices were considered secondary to portal hypertension due to extrahepatic portal vein obstruction. The patient was transferred to the pediatric intensive care unit because of hypovolemic shock caused by esophageal variceal bleeding. Subsequently, a gastroenterologist performed an upper gastrointestinal endoscopy under endotracheal intubation with respiratory support. The endoscopy confirmed the presence of esophageal varices, which had been identified by CT. No active bleeding or identifiable source requiring hemostatic intervention was observed (Figures 4A, 4B, 4C).

The patient received four units of red blood cell (RBC) transfusion for anemia. On day three of hospitalization, she was successfully extubated. Peramivir was administered for four days. On day five of hospitalization, she resumed oral intake, and no further bleeding or progression of anemia was observed.

**Figure 5:**
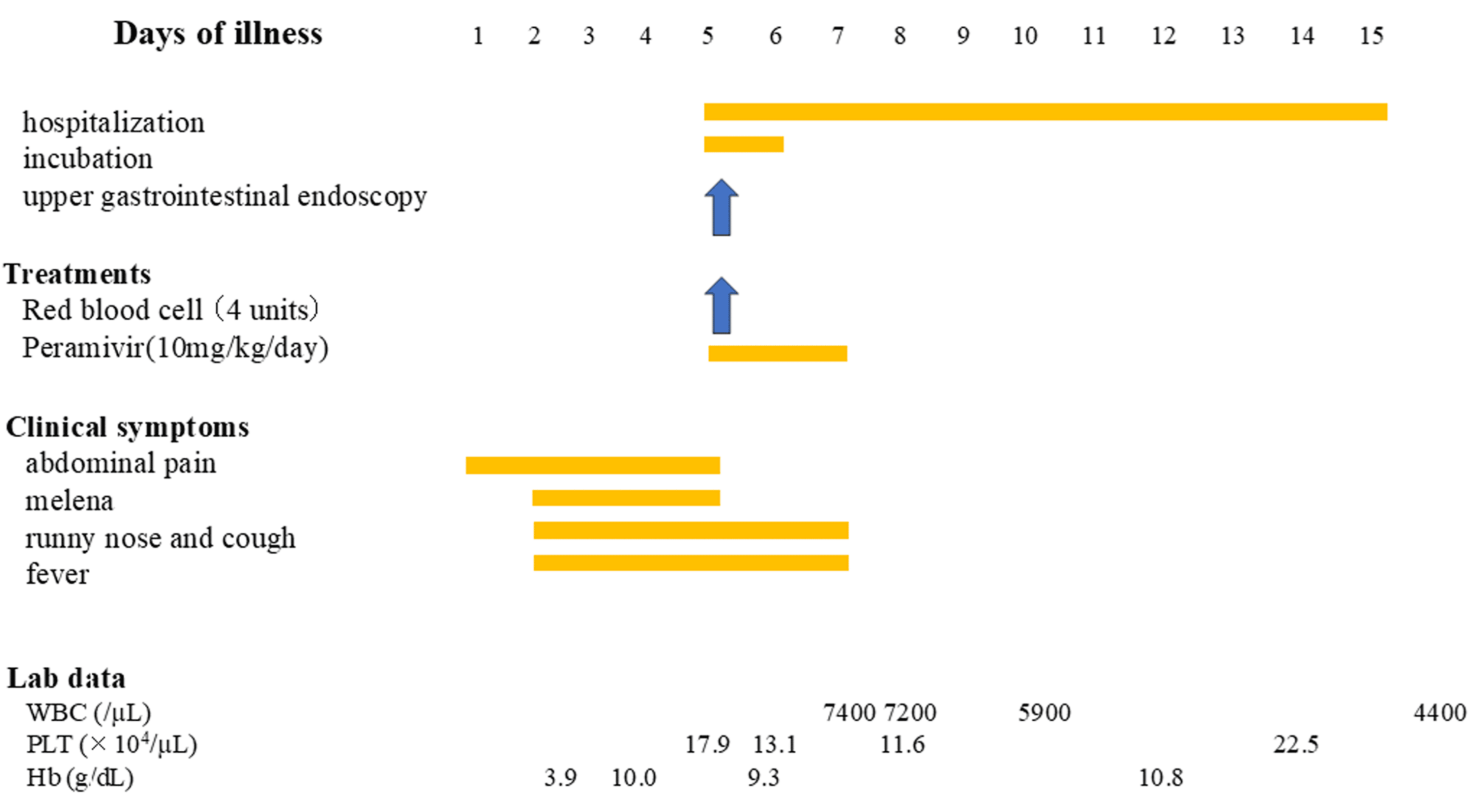
Clinical course On day four of illness, she presented to our hospital with abdominal pain, melena, and difficulty ambulating. CT revealed esophageal varices, and she was admitted on day five. Upper gastrointestinal endoscopy performed on day five confirmed the absence of active bleeding. On the same day, RBC transfusions and peramivir therapy were initiated. Her symptoms resolved by day seven. Laboratory findings showed recovery of Hb levels to 10.8 g/dL by day 14. On day 15, she was transferred to another hospital for evaluation for liver transplantation. Parameters (reference ranges): white blood cells (4200-18300/μL), Plts (18.0-58.0×10⁴/μL), Hb (11.1-14.2 g/dL). RBC: red blood cell; CT: computed tomography; Hb: hemoglobin; Plt: platelet; WBC: white blood cell

Esophageal varices indicated the presence of portal hypertension. To prevent complications related to chronic portal hypertension, a Meso-Rex bypass or, in end-stage cases, liver transplantation may be necessary [12]. On day 12 of hospitalization, the patient was discharged and referred to another medical facility to prepare for further treatment (Figure 5).

## Discussion

This patient was diagnosed with LAI at the age of four years following hypovolemic shock caused by esophageal variceal bleeding, despite having previously undergone cardiac catheterization and intracardiac repair. She exhibited a duplicated IVC, a venous anomaly that is rarely associated with AVSD.

**Figure 6:**
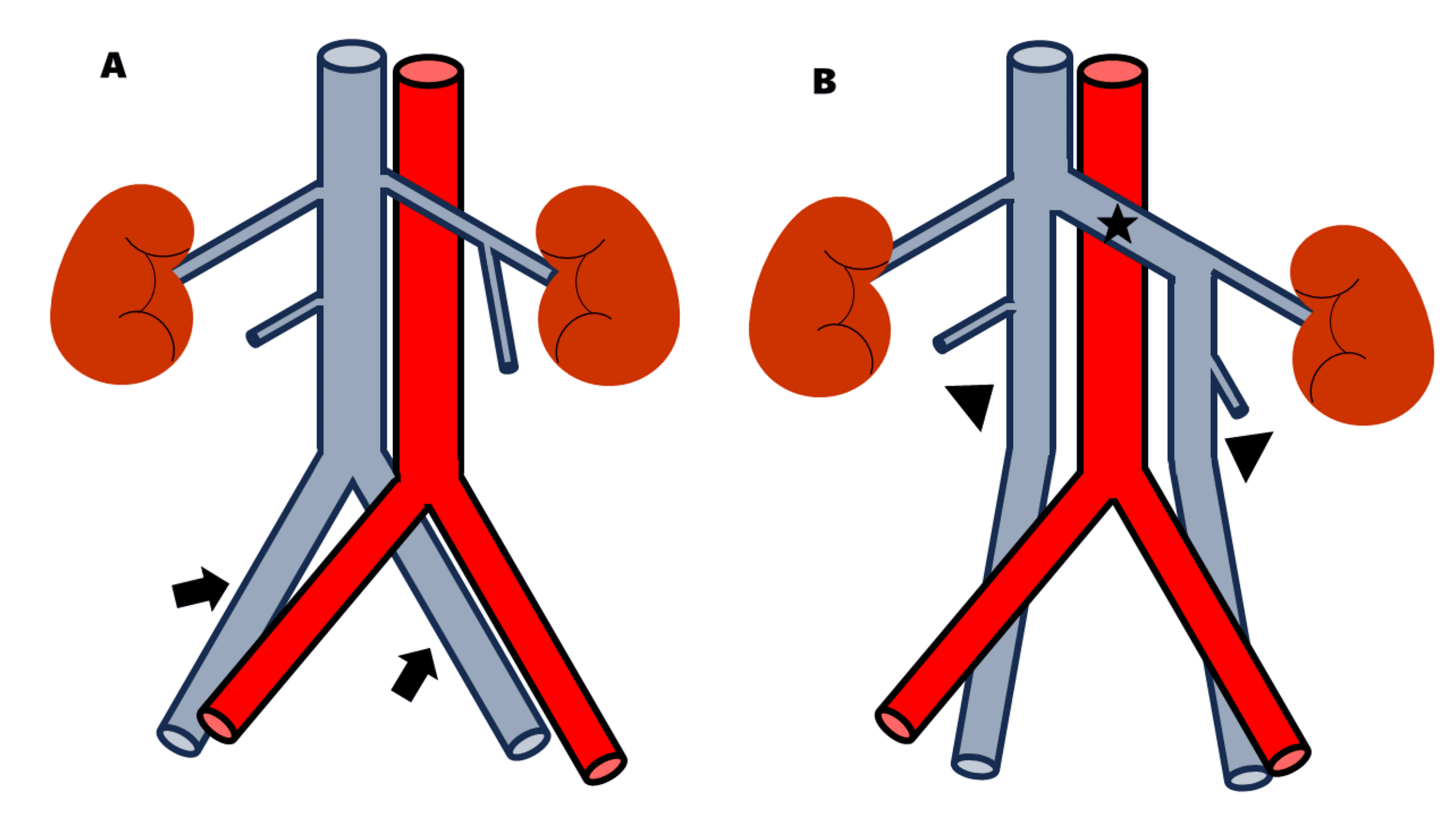
Venous anatomy in duplicated IVC A: In normal anatomy, the left and right common iliac veins (arrows) converge to form the IVC. B: In duplicated IVC anatomy, the left and right IVCs (arrowheads) ascend on either side of the abdominal aorta. The left IVC crosses anterior to the aorta at the level of the renal veins (star) to join the right-sided IVC. IVC: inferior vena cava Image Credit: Authors, using Microsoft PowerPoint (Microsoft Corporation, Redmond, Washington, USA)

In a duplicated IVC, the common iliac veins fail to unite. In the common variant, the left and right IVCs ascend on either side of the abdominal aorta. The left IVC crosses anterior to the aorta at the level of the renal veins to join the right-sided IVC (Figure 6) [13]. A duplicated IVC originates from an embryological anomaly. During embryonic development, both the left and right supracardinal veins arise. In normal development, the left supracardinal vein disappears, while the right supracardinal vein persists and forms the IVC [14]. Persistence of both supracardinal veins leads to the development of a duplicated IVC up to the level of the left renal vein [15]. The left IVC then joins the left renal vein and continues to the right to drain into the right IVC. The incidence of a duplicated IVC ranges from 0.3-3.0% in the general population [15,16]. To our knowledge, an association between LAI and a duplicated IVC has not been reported, whereas IVC defects are observed in 78% of patients with LAI [17].

In this patient, there was a merged IVC that drained into the right atrium. The absence of an IVC defect made the diagnosis of LAI difficult. We did not reach a diagnosis of a duplicated IVC on routine echocardiography, which revealed only drainage of the IVC into the right atrium. Additional ultrasonography of the upper abdomen, focusing on the abdominal venous system, may have helped detect the duplicated IVC.

LAI had not been detected even after cardiac catheterization prior to intracardiac repair for AVSD. The right femoral vein is normally used as the venous access route in pediatric cardiac catheterization, whereas the left femoral vein is not typically used when right femoral venous access is successful [18]. If the left femoral vein had been used as a venous access site, the abnormal abdominal venous system and the drainage of the left IVC into the right IVC near the liver might have been detected. In this patient, the left and right IVCs merged near the liver, which differs from the normal finding in which the left and right iliac veins unite to form a single right-sided IVC at a more caudal level [19].

The patient tested positive for influenza A, which may have been related to the esophageal variceal bleeding. A previous study examining the elevation of central venous pressure (CVP) during coughing reported that it may reach up to 60 cm H₂O [20]. In addition, severe coughing has been shown to increase intra-abdominal and variceal pressures, potentially precipitating rupture of esophageal varices [21]. Elevation of intra-abdominal pressure and CVP increases venous pressure in downstream veins, including esophageal varices [22]. Therefore, the transient elevation in venous pressure induced by coughing may have contributed to rupture of the esophageal varices.

Thorough abdominal ultrasonography may be important in patients with AVSD to detect abdominal complications [23]. Polysplenia and intestinal malrotation are important clues for the diagnosis of LAI on ultrasonography [8,9]. However, to date, no study, to our knowledge, has examined the sensitivity of ultrasonography for diagnosing polysplenia or intestinal malrotation. Moreover, the sensitivity largely depends on the type of abnormality present and the skill and experience of the ultrasonographer. It is imperative to develop more sensitive and widely applicable methods for detecting heterotaxy.

Our literature search did not identify any studies reporting the prevalence of duplicated IVC in patients with heterotaxy. Nevertheless, this venous variant has the potential to indicate systemic anatomical abnormalities. Thus, in patients with a duplicated IVC, heterotaxy should be considered in the differential diagnosis, especially in those with AVSD.

In our patient, liver enzyme levels were within normal limits, and she did not exhibit clinical symptoms indicative of portal hypertension before developing esophageal variceal bleeding. Therefore, it would have been difficult to predict hypovolemic shock even if she had been diagnosed with LAI at an earlier stage. On the other hand, in patients with heterotaxy, including LAI, comprehensive evaluation for systemic comorbidities using imaging modalities has been recommended [24]. Earlier recognition of heterotaxy might have prompted more careful evaluation for extracardiac comorbidities, including hepatic disease.

This case highlights the importance of comprehensive structural evaluation in patients suspected of having LAI. Recognition of abnormal venous anatomy may provide an opportunity for early detection of portal venous obstruction or LAI, potentially allowing early management before the development of life-threatening complications.

## Conclusions

In the case of this four-year-old girl, LAI was ultimately diagnosed after an episode of esophageal variceal bleeding, despite her having previously undergone intracardiac repair for AVSD. This late diagnosis resulted from the absence of the cardiovascular defects typically seen in LAI and the fact that her abdominal abnormalities had been overlooked. Of note, the patient exhibited duplicated IVCs, which are rarely associated with LAI but may indicate the presence of LAI. To enable a diagnosis of heterotaxy before the onset of fatal complications, further accumulation of cases with detailed information on venous variants, as well as the establishment of a systematic diagnostic approach, is warranted in future studies.
